# Functional Characterization of Two Low-Density Lipoprotein Receptor Gene Mutations in Two Chinese Patients with Familial Hypercholesterolemia

**DOI:** 10.1371/journal.pone.0092703

**Published:** 2014-03-26

**Authors:** Haihong Wang, Shengyuan Xu, Liyuan Sun, Xiaodong Pan, Shiwei Yang, Luya Wang

**Affiliations:** Department of Atherosclerosis, Beijing Institute of Heart Lung and Blood Vessel Disease, Beijing Anzhen Hospital Affiliated with Capital Medical University, Beijing, China; Cardiological Center Monzino, Italy

## Abstract

**Background:**

Familial hypercholesterolemia (FH) is an autosomal dominant disease that primarily results from mutations in the low-density lipoprotein receptor (*LDLR*) gene. We investigated two unrelated Chinese FH patients using gene screening and functional analysis to reveal the pathogenicity and the mechanism by which these mutations cause FH.

**Methods:**

First, the *LDLR* gene was sequenced in these patients. Then, mutant receptors were transfected into human embryo kidney 293(HEK-293) cells, and a confocal laser-scanning microscope was used to observe the localization of mutant proteins. Further, the expression and the internalization activity were analyzed by flow cytometry. Finally, LDLR protein expression and stability was detected by western blot.

**Results:**

Two different *LDLR* class 2B mutations were detected in two patients. The C201F mutation is a known mutation. However, the G615V mutation is novel. Flow cytometry showed that the expression and internalization activity of the mutant LDLRs were reduced to 73.6% and 82.6% for G615V and 33.2% and 33.5% for C201F, respectively.

**Conclusions:**

This study identified two *LDLR* mutations in Chinese patients with FH and analyzed the relationship between the genotype and phenotype of these patients. We found that these mutant LDLRs were defective in transport, which led to a reduction in cholesterol clearance. These results increase our understanding of the mutational spectrum of FH in the Chinese population.

## Introduction

Familial hypercholesterolemia (FH) is an autosomal dominant, inherited disease of lipid metabolism. Clinical manifestations of FH are abnormally high concentrations of plasma low-density lipoprotein cholesterol, tendon xanthomas and premature coronary heart disease. Total plasma cholesterol concentrations in heterozygous FH patients are typically in the range of 7 to 15 mmol/L and in homozygous FH range from 20 to 25 mmol/L. FH heterozygotes present manifestations of CHD at the age of 30–40 years, and homozygous FH present CHD before the age of 20 years [1] [2]. It is largely believed that in the general population, the prevalence of the homozygous FH is 1/1,000,000, while the prevalence of heterozygous FH is 1/500 [3]–[5]. However, in the Copenhagen General Population study, the prevalence of FH was approximately 1/200 [6]. Based on these estimates of prevalence, there are approximately 14–34 million individuals with FH worldwide [7]. Prevalence of FH might be as high as 1/80 in some populations, especially in French Canadians [8]. FH is commonly caused by mutations in the low-density lipoprotein receptor (*LDLR*), apolipoprotein B (*apoB*), or proprotein convertase subtilisin/kexin type 9 (*PCSK9*) genes. In particular, *LDLR* gene mutations are the most frequent cause of FH [9].

The LDLR is a cell-surface glycoprotein responsible for the uptake and removal of cholesterol-rich lipoproteins particles from the circulation [10]. According to biochemical and functional studies of *LDLR*, *LDLR* mutations can be divided into five classes. Class 1 mutations include null alleles with no detectable LDLR protein. Class 2 mutations encode LDLR proteins with defective transport from the endoplasmic reticulum to the Golgi apparatus that is either completely (class 2A) or partially blocked (class 2B).Class 3 mutations produce LDLR proteins that are defective in binding LDL. Class 4 mutations encode LDLR that fails to internalize LDL. Finally, class 5 mutations produce recycling-defective receptors [11]–[13]. To date, more than 1,200 *LDLR* mutations have been documented worldwide [14]. However, it has been previously shown that the presence of a mutation in the *LDLR* does not necessarily result in FH occurrence [15], [16]. Therefore, there is a need for functional validation of *LDLR* mutations to determine their pathogenicity.

In this study, we investigated two unrelated Chinese FH probands and their first-degree relatives. One novel and one previously reported *LDLR* mutation were found. We further investigated the pathogenicity and the mechanism of these FH-causing mutations using a combination of transfection, confocal laser scanning microscopy, flow cytometry analysis, and western blot.

## Materials and Methods

### Patients

Two probands were recruited from the Beijing Anzhen Hospital that met the following FH clinical diagnostic criteria: 1) for adults, total cholesterol (TC) >7.8 mmol/L or low-density lipoprotein cholesterol (LDL-C) >4.4 mmol/L; 2) for children younger than 16 years old, TC >6.7 mmol/L; 3) patients or their relatives have tendon xanthomas; 4) patients with xanthomas whose TC is higher than 16 mmol/L are diagnosed as homozygous and the others patients are diagnosed as heterozygous [17].

### Ethics Statement

The study was reviewed and approved by the ethical committee of the Beijing Anzhen Hospital, and all participants signed an informed consent form. The written informed consent for the minor enrolled in the study was obtained from his guardian.

### Blood Lipid Measurements

Peripheral venous blood samples from the two probands and their first-degree relatives were drawn after a 12-hour fast. TC, LDL-C, triglycerides (TG) and high-density lipoprotein cholesterol (HDL-C) were measured using routine commercial kits (Beckman Coulter, Brea, USA) and an automated biochemistry analyzer (Beckman AU 4500, Brea, USA).

### Sequencing of *LDLR*, *APOB* and *PCSK9* Genes

Genomic DNA was extracted using the phenol-chloroform centrifugation method. Then, the coding regions of *LDLR* (containing the promoter and 18 exons with flanking intron sequences), and *PCSK9* (containing the 12 exons with flanking intron sequences) genes as well as part of exon 26 of the APOB gene, associated with the apoB -LDLR interaction were amplified by polymerase chain reaction (PCR). The sequences of oligonucleotide used for the amplification are shown in the Table S1, S2 and S3. Thereafter, the amplification products were purified and then sequenced on ABI Prism 3730×l DNA Analyzer. The results were analyzed by phred/phrap/consed package. Finally, when mutations were detected, another sequencing reaction was performed on both genomic DNA from the relative and on a new PCR product from the proband.

### Construction of a *LDLR* Mutant

We cloned the wild-type *LDL-R* gene from the hepatocyte cell line BEL 7402 in the OmicsLink (Guangzhou FulenGen, China) mammal cell expression vector, which has an N-terminal enhanced green fluorescent protein (EGFP) tag. The *LDLR* mutations (G615V and C201F) were generated from OmicsLink-LDLR by oligonucleotide site-directed mutagenesis using a QuikChange XL mutagenesis kit (Stratagene) according to the manufacturer’s instructions. The following oligonucleotides were used to generate different plasmids carrying the *LDLR* mutations:

5′-TGCCAACCGCCTCACAG**T**TTCCGATGTCAACTTGT-3′ (mutated base in bold) was used to change the codon GGT, which encodes glycine (G615), to GTT, which encodes valine (V);

5′-TGGTGGCCCCGACT**T**CAAGGACAAATCTG-3′ (mutated base in bold) was used to change the codon TGC, which encodes cysteine (C201), to TTC, which encodes phenylalanine (F).

The integrity of all of the constructs was confirmed by direct sequence analysis.

### Cell Culture and Transfection

Human embryonic kidney 293 (HEK-293) cells, which do not express LDLR [18], were cultured in Dulbecco’s modified Eagle’s medium (DMEM) supplemented with 10% fetal bovine serum, 100 U/ml penicillin and 100 μg/ml streptomycin at 37°C in a humidified atmosphere of 5% CO_2_. In total, 3×10^5^ cells were seeded in 6-well plates for 24 hours and plasmids containing wild-type and mutant *LDLR* were transfected into HEK-293 cells using lipofectamine2000 reagent (Invitrogen) according to the manufacturer’s instructions. Transfection efficiency was estimated by counting the EGFP positive cells under a fluorescent microscope. Confocal laser scanning microscope and flow cytometry were used to determine the function of the LDLR following a 48-hour culture.

### Confocal Laser Scanning Microscope (CLSM)

To determine the cellular localization of the LDLR-EGFP fusion proteins and to analyze the LDLR activity by measuring internalization of LDL in transfected cells, CLSM (Leica) was used. To obtain the fluorescent images, a 63×oil objective was used.

To observe the cellular localization of the LDLR-EGFP fusion proteins, the cells transfected with wild-type protein, G615V or C201F LDLR were grown on cover slips, fixed and permeabilized with 70% ethanol for 10 minutes. Then, the cells were incubated in PBS containing 1 ml/L Triton X-100 for 15 minutes at room temperature. Afterwards, the cells were washed three times in PBS and incubated with tetramethylrhodamine-conjugated concanavalin A (1∶100, Molecular Probes) at room temperature for 1 hour. Next, the cells were washed three times with PBS and fixed with 4% paraformaldehyde for 15 minutes at room temperature. Finally, the intracellular fluorescent dye was observed by CLSM.

To analyze the LDLR activity, the cells transfected with wild-type, G615V or C201F LDLR were grown on cover slips and incubated in serum-free media containing 20 μg/ml fluorescent 1,1′-dioctadecyl-3,3,3′3′-tetramethylindocarbocyanine perchlorate (Dil)-conjugated LDL (Molecular Probes) for 4 hours at 37°C. Following the LDL incubation, the medium was removed, and the cells were washed three times with PBS and fixed with 4% paraformaldehyde for 15 minutes at room temperature. Finally, the intracellular fluorescent dye was observed by CLSM.

### Flow Cytometry Analysis

Flow cytometry was used to detect the amount of cell-surface LDLR expression and LDL internalization. The fluorescence of 10,000 events for each sample was acquired for data analysis. Forward scatter versus side scatter gates were set to exclude dead cells and debris. Experiments were repeated at least three times with triplicate samples for each cell line.

To measure the amounts of cell-surface LDLR expression, the cells transfected with wild-type, G615V or C201F LDLR were harvested 48 hours after transfection and resuspended in PBS containing 1% BSA. Then, the cells were washed twice in PBS containing 1% BSA and incubated with phycoerythrin (PE)-conjugated mouse monoclonal anti-human LDLR (1∶20, R&D) at room temperature for 30 minutes in the dark. Finally, the cells were washed three times with PBS containing 0.1% BSA and resuspended in PBS. PE fluorescence was quantified using a FACS Calibur (Beckman Coulter).

To measure LDLR internalization activity, the cells transfected with wild-type, G615V or C201F LDLR were incubated in serum-free media containing 20 μg/ml Dil-LDL for 4 hours at 37°C. Following the LDL incubation, the medium was removed, and the cells were detached from the culture dish. Afterwards, the cells were washed twice in PBS and resuspended in PBS. The amount of LDL internalization was detected using a FACS Calibur (Beckman Coulter).

### Western Blot

Western blot was used to investigate LDLR protein expression levels and stability. The protein contens were normalized to the level of GADPH.

To measure the LDLR protein expression levels and stability, the cells transfected with wild-type, G615V or C201F LDLR were harvested 48 hours after transfection. Fresh cells were harvested with lysis buffer. Protein samples (30 μg) were separated by 8% SDS-PAGE, transferred onto nitrocellulose membranes for incubated with the primary antibodies anti-LDLR (1∶500, Abcam, Cambridge, UK) or anti-GADPH (1∶3000 diluted in TBS-T, Kangwei, China) at 4°C overnight, then with IR Dye-conjugated secondary antibodies (1∶5000, Rockland Immunochemicals, Gilbertsille, PA) for 1 hour. Images were quantified by use of the Odyssey infrared imaging system (LICOR Biosciences Lincoln, NE, USA). All experiments were repeated at least 3 times.

## Results

### Clinical Features

The blood lipid concentrations of the two probands and their first-degree relatives are shown in the Table 1. The data was collected for the first time in our experiment. The patients had received some lipid-lowering drugs therapy before our experiment.

**Table 1:** Clinical features and gene identification results of the probands and their first relatives.

Pedigree	Patient	Sex	Age	TC (mmol/L)	LDL-C (mmol/L)	TG (mmol/L)	HDL-C (mmol/L)	Nucleic acid change	Mutation
I	Proband1	Female	40	16.87	13.93	2.47	0.73	c.1907G>T	p.G615V
	Aunt	Female	58	4.93	3.10	1.40	1.05	c.1907G>T	p.G615V
	Mother	Female	67	8.29	5.71	1.42	1.41	normal	normal
	Sister	Female	46	8.91	5.39	0.98	1.27	c.1907 G>T	p.G615V
	Son	Male	17	4.62	2.83	1.23	1.95	normal	normal
II	Proband2	Male	13	12.79	10.05	1.50	1.19	c.665G>T	p.C201F
	Father	Male	35	5.71	2.69	3.93	0.97	c.665G>T	p.C201F
	Mother	Female	34	6.37	3.61	1.77	2.16	normal	normal

Proband 1 presented with multiple xanthomas on the bilateral eyelids, extensor tendon and buttocks when the patient was 8 years old. B-mode Doppler ultrasound revealed increased intima-media thickness and multiple atherosclerotic plaques in the common carotid arteries, the bilateral femoral arteries, and the bilateral external iliac arteries. Transthoracic Doppler echocardiography showed that each cardiac chamber was within the normal range and the thickness and motion of each ventricular wall was normal, but the coronary flow velocity reserve (CFVR) in the distal left anterior descending coronary artery (LAD) was reduced to 1.9 (normal value ≥3).

At the age of four years old, proband 2 had two cutaneous xanthomas on his buttocks that gradually increased. In the following years, multiple cutaneous xanthomas were noticed on the bilateral eyelids, hands, elbows and knees. Tendon xanthomas were also discovered on the extensor tendons over the Achilles and the knees. The interphalangeal joints were widened and deformed, the Achilles tendons were widened, and the cornea arcus was apparent. The electrocardiogram indicated a more than 0.05 mV depression in the ST-segment in I, avL, V5 and V6. Transthoracic Doppler echocardiography showed the following: the aorta wall was thickened and the lumen was narrowed, the whole heart was enlarged, and systolic function of the heart was obviously decreased. Mild mitral regurgitation, hydropericardium, valve thickening, and left and right coronary artery diffuse stenosis were also discovered. The ejection fraction (EF) was decreased to 25.5%, and the CFVR was reduced to 1.16.

### Identification of Gene Mutations

The *LDLR* mutations found in the two probands and their first-degree relatives are shown in the Table 1. No mutations in *apoB* and *PCSK9* genes were detected in these patients.

**Figure 1:**
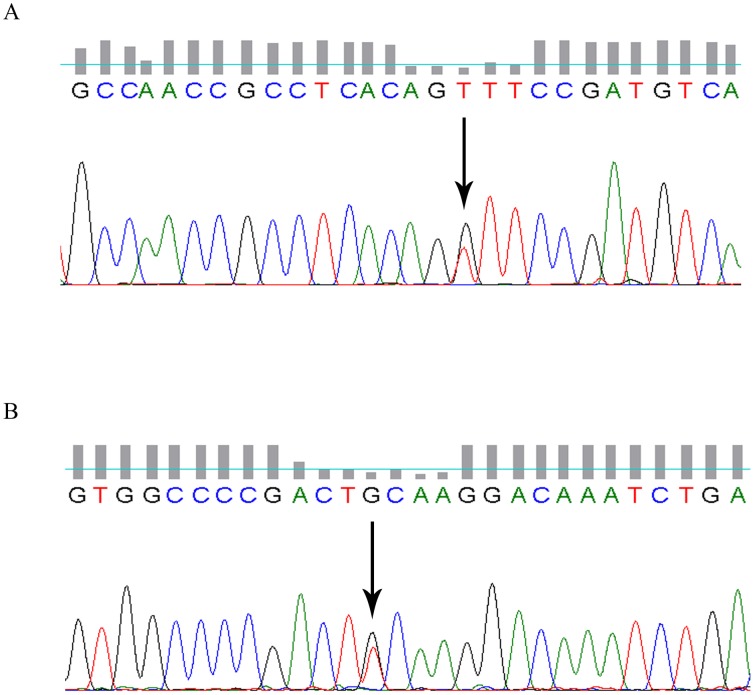
The DNA sequencing results of the two FH probands. (A) The *LDLR* gene of proband 1. The arrow indicates the G>T missense mutation at position 1907 of the thirteenth exon resulting in a glycine to valine substitution; (B) The *LDLR* gene of proband 2. The arrow indicates the G>T missense mutation at position 665 of the fourth exon resulting in a cysteine to phenylalanine substitution.

For proband 1, a single base substitution (G>T) at position 1907 in the thirteenth exon of the *LDLR* gene was identified, which leads to a change from glycine (GGT) to valine (GTT) in the protein sequenced at position 615 (Figure 1A). We were unable to obtain blood from the proband’s father and therefore do not have the gene sequence for the *LDLR* from the proband’s father. However, we did sequence the *LDLR* from the proband’s paternal aunt. Interestingly, the same heterozygous missense mutation at G615V was detected in the proband’s aunt. In addition, the proband’s sister was also identified as having the G615V mutation. The *LDLR* locus of the proband’s mother and son was normal.

Proband 2 had a single base substitution (G>T) in exon 4 of the *LDLR* gene at nucleotide 665, which changes codon 201 in the mature protein from cysteine (TGC) to phenylalanine (TTC) (Figure 1B). The same heterozygous missense mutation was detected in the proband’s father. Proband 2′s mother was not identified as having the C210F mutation.

### Confocal Laser Scanning Microscopy Analysis of Wild-type and Mutant LDLR in HEK-293 Cells

**Figure 2:**
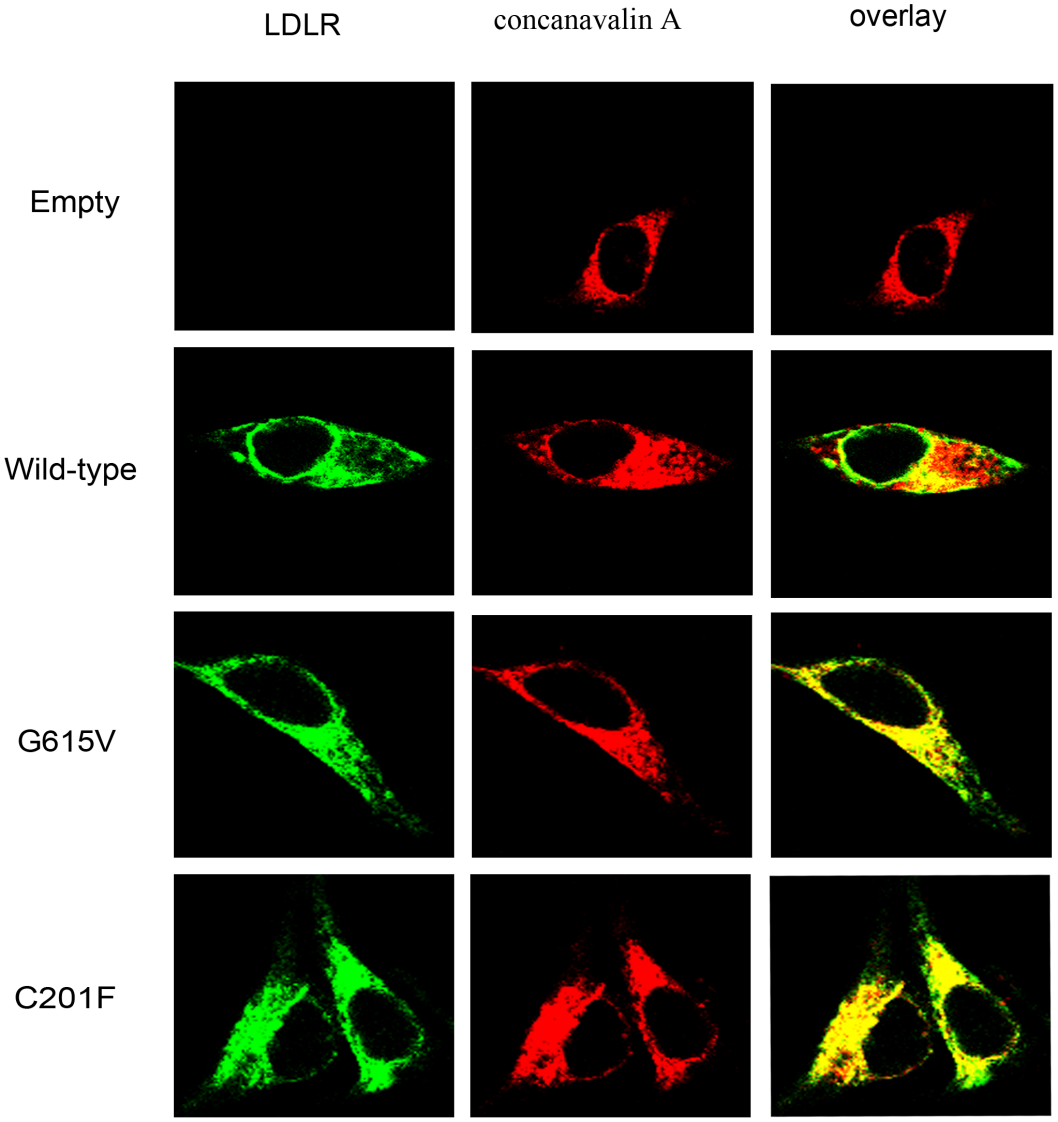
Confocal laser images of wild-type, G615V and C201F LDLR localization in transfected HEK-293 cells. Cells were incubated with tetramethylrhodamine-conjugated concanavalin A at room temperature for 1 hour. Overlays are shown in the right panels with co-localization appearing yellow. Similar results were obtained in 3 separate experiments.

The localization of LDLR was investigated using co-localization of LDLR and tetramethylrhodamine-conjugated concanavalin A. Concanavalin A specifically binds to mannose-rich glycans in the endoplasmic reticulum (ER) [19]. The results showed that wild-type LDLR was arranged as a circle and was primarily distributed on the cell surface. However, the LDLR accumulated in the cytoplasm for G615V and C201F (Figure 2). Therefore, these two mutations are classified as class 2 mutations. For simplicity, only a single cell is shown in Figure 2, but this cell is representative of the majority of the cells scanned in each field.

**Figure 3:**
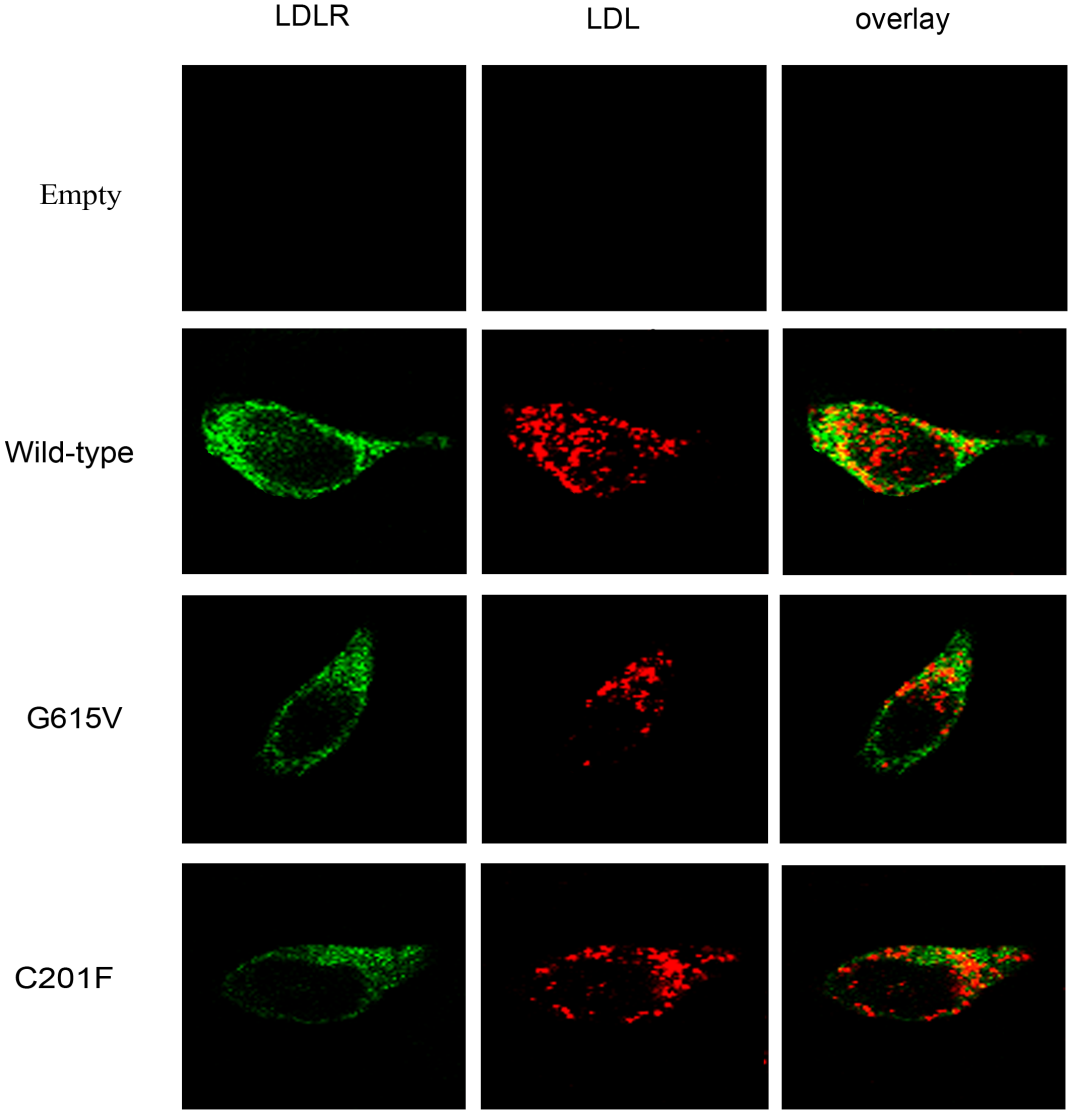
Confocal laser images of wild-type, G615V and C201F LDLR activity in transfected HEK-293 cells. Cells were incubated with 1,1′-dioctadecyl-3,3,3′3′-tetramethylindocarbocyanine perchlorate (Dil)-conjugated LDL for 4 hours at 37°C. Overlays are shown in the right panels with co-localization appearing yellow. Similar results were obtained in 3 separate experiments.

LDLR function was analyzed by evaluating the cells ability to internalize Dil-LDL. The results showed that both G615V and C201F LDLR mutants still retained internalization function (Figure 3). Combined with the above results, we conclude that the two mutations belong to class 2B mutations. Surface expression and activity of the LDLR was further investigated by flow cytometry.

### Flow Cytometry Analysis of Cell-surface LDLR Expression and LDL Internalization in HEK-293 Cells

**Figure 4:**
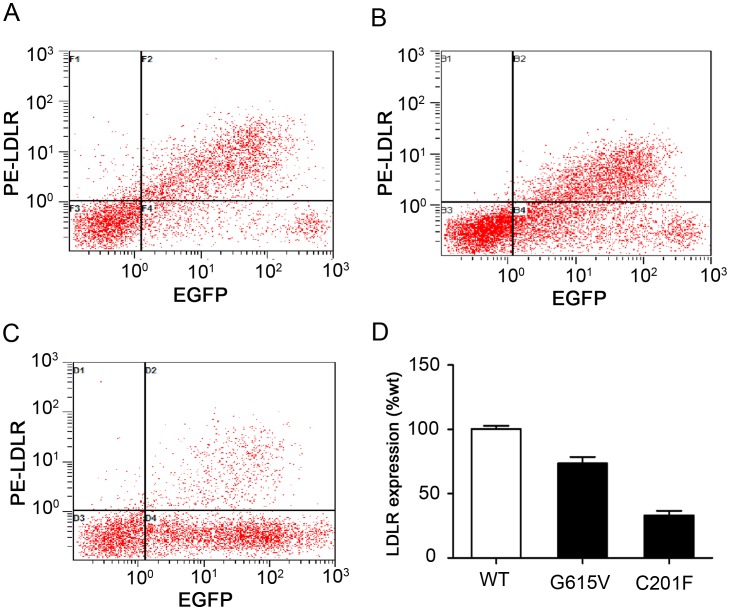
Flow cytometric measurements of wild-type and mutatant LDLR expression in transfected HEK-293 cells. The cells were incubated with phycoerythrin (PE)-conjugated mouse monoclonal anti-human LDLR antibody at room temperature for 30 minutes. The upper right area of the dot plots represents EGFP and LDLR double positive cells. (A) Transfected with wild-type; (B) Transfected with the G615V mutant LDLR; (C) Transfected with the C201F mutant LDLR; (D) The histogram shows the percentage of fluorescence for each of the mutations relative to wild-type. The results are representative of the means ± SD for three independent experiments.

**Figure 5:**
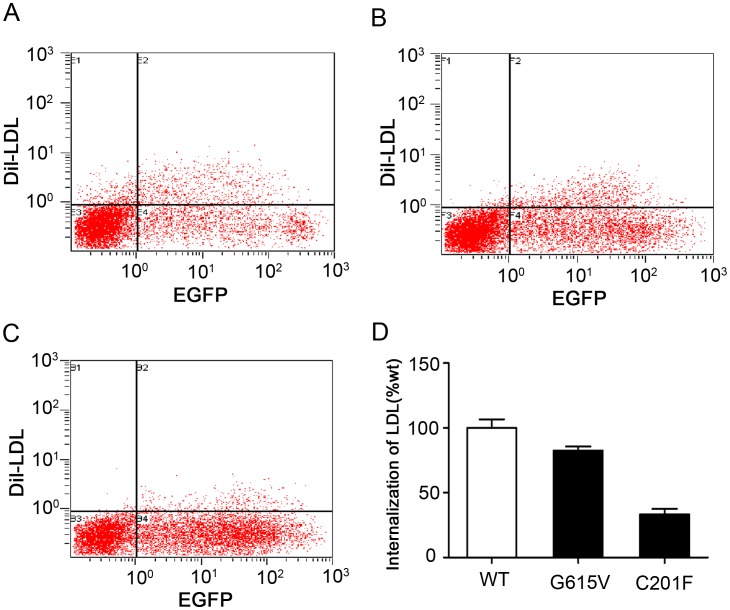
Flow cytometric measurements of wild-type and mutant LDLR internalization activity in transfected HEK-293 cells. The transfected cells were incubated in serum-free media containing 20 μg/ml Dil-LDL at 37°C for 4 hours. The upper-right area of the dot plots represents EGFP and LDLR double positive cells. (A) Transfected with wild-type; (B) Transfected with the G615V mutant LDLR; (C) Transfected with the C201F mutant LDLR; (D) The histogram shows the percentage of fluorescence for each of the mutations relative to wild-type LDLR. The results are representative of the means ± SD for three independent experiments.

Flow cytometry was used to detect cell-surface LDLR expression and LDL internalization in HEK-293 cells that were transfected with wild-type or mutant LDLR (G615V and C201F). This analysis revealed that the cell-surface expression level of G615V and C201F LDLR were reduced to 73.6% and 33.2%, respectively, when compared with wild-type LDLR (Figure 4). Additionally, it was observed that both mutations impaired the ability of the LDLR to internalize LDL. Cells expressing G615V and C201F mutant receptors showed 82.6% and 33.5% residual activity, respectively, when compared to cells expressing wild-type LDLR (Figure 5).

### Western Blot Analysis of LDLR Protein Expression and Stability in HEK-293 Cells

**Figure 6:**
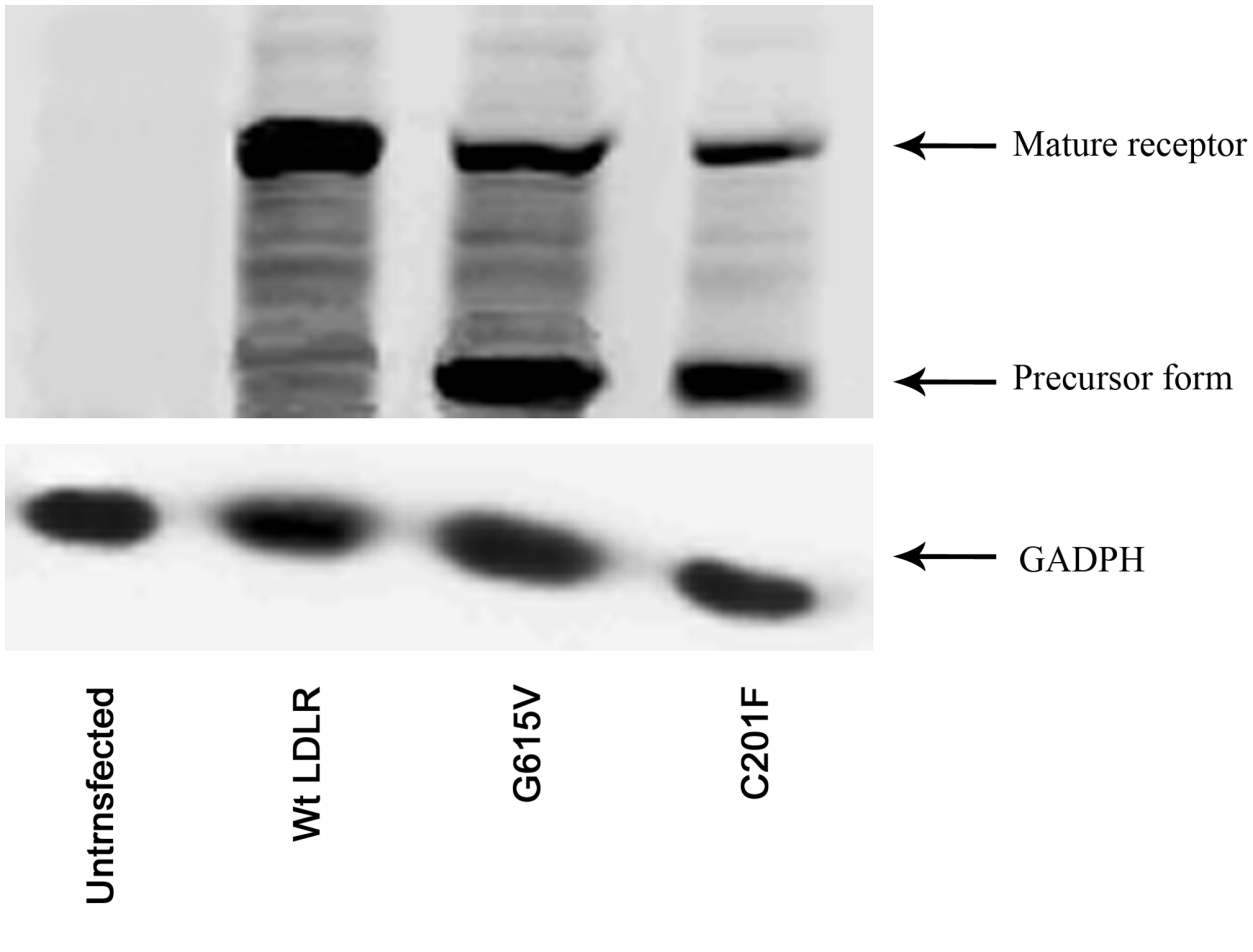
Western blot analysis of wild-type and mutant LDLR protein expression in transfected HEK-293 cells. Whole cell extracts (30 μg) were separated by 8% SDS-PAGE, transferred onto nitrocellulose membranes for incubation with a specific antibody against the LDLR. For wild-type LDLR only one band was detected, corresponding to the mature form. For mutant G615V and C201F two bands were detected, though the amount of mature protein was lower compared to the wild-type receptor and it could be seen a small amount of the precursor form, more evident in variant C201F.

Western blot was used to detect LDLR protein expression and stability in HEK-293 cells that were transfected with wild-type or mutant LDLR (G615V and C201F). Relative expression and the ability of those cells to express LDLR were assayed by immunoblotting with a specific antibody against the LDLR. For wild-type LDLR only one band was detected, corresponding to the mature form (apparent molecular weight ∼160 kDa). For mutant G615V and C201F two bands were detected, though the amount of mature protein was lower compared to the wild-type receptor and it could be seen a small amount of the precursor form (apparent molecular weight ∼120 kDa) of the receptor protein, more evident in variant C201F (Figure 6).

## Discussion

In the current study, two different *LDLR* gene mutations, G615V and C201F, were identified in two probands. In particular, the G615V mutation has not been reported elsewhere, while the C201F was previously found in the Netherlands [20]. However, there is no data on the *in vitro* functional consequence of the C201F mutation.

We used an *in vitro* experiment to identify the functional consequences of the *LDLR* mutations. Flow cytometry showed that the expression of cell-surface LDLR was reduced to 73.6% and 33.2% for the G615V and C201F LDLR mutations, respectively, relative to wild-type LDLR. CLSM showed that mutant proteins partially accumulated in the endoplasmic reticulum. The results of western blot showed that the amount of mature protein detected for mutants G615V and C201F was lower compared to the wild-type receptor and it could be seen a small amount of the precursor form. Therefore, both G615V and C201F mutations are classified as class 2B mutations. The incidence rate of class 2 mutations is more than 50%, which is the highest of all *LDLR* mutations that result in FH [11], [21]. The endoplasmic reticulum contains many folding chaperones and enzymes that promote the folding and transport of newly synthesized proteins. These chaperones and enzymes also identify proteins unable to fold correctly and hinder them from being exported from the ER to the Golgi apparatus [19]. The accumulation of misfolded proteins in the ER can cause ER stress.

However, a portion of the mutant proteins were able to reach the cell surface and retain their function despite the G615V and C201F LDLR mutants being partially retained in the ER. Flow cytometry showed that LDL internalization was reduced to 82.6% and 33.5% for G615V and C201F LDLR mutants, respectively, relative to wild-type LDLR. Class 2 mutations are unable to remove cholesterol from the blood plasma, which eventually leads to the occurrence of atherosclerosis and coronary heart disease because the LDLR is unable to reach the cell-surface. FH has therefore been classified as an ER retention disease [22].

The G615V and C201F mutations are located in different functional domains of the LDLR. G615V is located in the epidermal growth factor precursor (EGFP) homology domain, which is 35% homologous to the EGFP. This domain contains two epidermal growth factor precursor like (EGFP-like) motifs followed by a series of six YWTD (tyrosine-tryptophan-threonine-aspartate) repeats and a third EGF-like motif [10]. The YWTD repeats form a six-bladed β-propeller. Approximately 54% of all *LDLR* gene mutations that result in FH occur in the EGFP homology domain [23], which means that this domain plays an important role in the function of the LDLR. The EGFP homology domain controls lipoprotein release in low pH environments and the recycling of the receptor back to the cell surface [24]. After the receptor-ligand complexes enter the cell by endocytosis, the low pH environment of the endosome causes the LDLR to change conformation and release its ligand completing the transport process. Under neutral pH conditions at the cell surface, the LDLR extends itself in an elongated form to bind LDL due to the function of the β propeller; however, in the acidic environment of the endosomes, the LDLR folds back on itself as the β propeller and competes with LDL for binding to the ligand-binding site [25], which results in LDL being released. The G615V mutation is located in the sixth YWTD repeats of the β propeller and likely affects the function of the β propeller. Therefore, the release of LDL and the recycling of the receptor back to the cell surface are impaired, which eventually leads to FH.

The C201F mutation is located in the ligand-binding domain, which contains seven LDL receptor type A (LA) repeats that are rich in cysteine. This domain is responsible for binding lipoproteins [10]. Some studies have shown that deleting the first two LA repeats has little influence on the binding of LDL. However, deletion of any other individual LA repeat leads to more than a 50% reduction in LDL binding [26], [27]. Therefore, the above results suggest that the LA repeats play a crucial role in binding LDL. The C201F mutation is located in the fifth LA repeat (LA5). Therefore, this mutation most likely affects LDL binding, which results in FH.

Although the LDLR activity of proband 1 was higher than that of proband 2, proband 2 had a lower level of plasma cholesterol compared to proband 1. For proband 1, the plasma TC level was 16.87 mmol/L and LDL-C was 13.93 mmol/L. For proband 2, the plasma TC level was 12.79 mmol/L and LDL-C was 10.05 mmol/L. However, in terms of the clinical phenotype of the target organ, proband 2 had a more serious phenotype than proband 1. For proband 2, the interphalangeal joints were widened and deformed, the Achilles tendons were widened. The aorta wall was thickened and the lumen was narrowed, the whole heart was enlarged, and systolic function of the heart was obviously decreased. Mild mitral regurgitation, hydropericardium, valve thickening, and left and right coronary artery diffuse stenosis were also discovered, the CFVR was reduced to 1.16. For prond 1, each cardiac chamber was within the normal range and the thickness and motion of each ventricular wall was normal, but the CFVR was reduced to 1.9.We speculate that the discrepancy between phenotype and LDLR activity in these two probands with G615V and C201F mutations may partly be influenced by other genetic and/or environment factors.

The mother of proband 1 was clinically diagnosed with heterozygous FH, but no *LDLR* gene mutation had been detected in this woman. Approximately 10%–40% of patients with a clinical diagnosis of FH do not have their *LDLR* mutation investigated [28], [29]. It is possible that these patients present a polygenic basis for their LDL-C elevation without contributions from any of the classical FH genes [7]. The inconsistency between clinical phenotype and genetic testing should be further studied.

In conclusion, two *LDLR* heterozygous missense mutations, G615V and C201F, were found in this study. In particular, the G615V mutation was a novel mutation. Functional research was performed for both mutations. The results showed that the mutant LDLRs were transport defective. The level of expression of the mutant LDLR and LDL internalization were both reduced compared to wild-type LDLR. These two mutations likely result in the LDLR being retained in the ER, and thus, the LDLR was unable to successfully reach the cell surface to clear LDL leading to FH. Our research increases the mutational spectrum of FH in the Chinese population. Identifying the pathogenicity of the *LDLR* mutations is of great importance to determine the actual cause of hypercholesterolemia. Additionally, understanding the mechanisms by which these mutations result in FH could help to determine a suitable lipid-lowering therapy for each patient.

## Supporting Information

Table S1 **Sequences of oligonucleotide used for the amplification of** ***LDLR*** **gene.**
(DOCX)

Table S2 **Sequences of oligonucleotide used for the amplification of** ***PCSK9*** **gene.**
(DOCX)

Table S3 **Sequences of oligonucleotide used for the amplification of** ***apoB*** **gene.**
(DOCX)
