# Supplementary material for: Functional Characterization of Two Low-Density Lipoprotein Receptor Gene Mutations in Two Chinese Patients with Familial Hypercholesterolemia
Source: PLoS One. 2014 Mar 26;9(3):e92703. doi: 10.1371/journal.pone.0092703 (PMC3966815; doi:10.1371/journal.pone.0092703)
Supplement: Table S1 — Sequences of oligonucleotide used for the amplification of LDLR gene. (DOCX) [file pone.0092703.s001.docx]

Table S1. Sequences of oligonucleotide used for the amplification of *LDLR* gene

| Exon | Forward Primer | Reverse Primer | Size of PCR Product（bp） |
| --- | --- | --- | --- |
| Promoter-Exon1 | 5'-CCCAAATACAACAAATCAAGTCG-3' | 5'-AGATGCGGTCCCTCACCCT-3' | 629 |
| Exon2 | 5'-TTAGTTGGCAGGAAATAGACA-3' | 5'-AACAAGATAGAGGTGGTGGC-3' | 600 |
| Exon3 | 5'-GCCTCAGTGGGTCTTTCCT-3' | 5'-TTGTAATGCCTCCTGGTCAA-3' | 357 |
| Exon4 | 5'-GTTGGGAGACTTCACACGGTGATGG-3' | 5'-GGGGGAGCCCAGGGACAGGTGATAG-3' | 550 |
| Exon5 | 5'-CTGGGCTCAAGCAATCCTCC-3' | 5'-CGCCCTCTGGCTTCACAAAT-3' | 347 |
| Exon6 | 5'-TCCTTCCTCTCTCTGGCTCTCACAG-3' | 5'-GCAAGCCGCCTGCACCGAGACTCA C-3' | 180 |
| Exon7 | 5'-AGATCGTGCCATTACACTCCA-3' | 5'-CATGAAACCCTCCTAACTGCTT-3' | 480 |
| Exon8 | 5'- CATTGGGGAAGAGCCTCCCC-3' | 5'-GCCTGCAAGGGGTGAGGCCG-3' | 220 |
| Exon9-10 | 5'-TCCATCGACGGGTCCCCTCTGACCC-3' | 5'-AGCCCTCAGCGTCGTGGATACGCAC-3' | 510 |
| Exon11 | 5'-AAAACCCAAACAAGCCACAT-3' | 5'-GGGAAACCTTCAGGGAGCAG-3' | 383 |
| Exon12 | 5'-GACCCTCTGGGACTGGCATCA-3' | 5'-CCTCCAGCCTGGGAAACGAG-3' | 389 |
| Exon13-14 | 5'-CCCAGTGTTTAACGGGATTT-3' | 5'-TACCCATTTGACAGATGAGCAG-3' | 560 |
| Exon15 | 5'-GAAGGGCCTGCAGGCACGTGGCACT-3' | 5'-GTGTGGTGGCGGGCCCAGTCTTT-3' | 250 |
| Exon16 | 5'-CCTTCCTTTAGACCTGGGCCT-3' | 5'-CATAGCGGGAGGCTGTGACC-3' | 170 |
| Exon17 | 5'-TGGCTCAACCTCGGCTCACT-3' | 5'-AGCGCACAGAAGCATTCACCTA-3' | 678 |
| Exon18 | 5'-GCCTGTTTCCTGAGTGCTGG-3' | 5'-TCTCAGGAAGGGTTCTGGGC-3' | 135 |
