# Supplementary material for: Functional Characterization of Two Low-Density Lipoprotein Receptor Gene Mutations in Two Chinese Patients with Familial Hypercholesterolemia
Source: PLoS One. 2014 Mar 26;9(3):e92703. doi: 10.1371/journal.pone.0092703 (PMC3966815; doi:10.1371/journal.pone.0092703)
Supplement: Table S2 — Sequences of oligonucleotide used for the amplification of PCSK9 gene. (DOCX) [file pone.0092703.s002.docx]

Table S2. Sequences of oligonucleotide used for the amplification of *PCSK9* gene

| Exon | Forward Primer | Reverse Primer | Size of PCR Product（bp） |
| --- | --- | --- | --- |
| Exon1 | 5'-TGCACAGTCCTCCCCACC-3' | 5'-AAGCGAAGAGCCCTCGG-3' | 439 |
| Exon2 | 5'-GGTCCGCATTTGGTAAC-3' | 5'-TTGCTGTCCCCTTCTGA-3' | 447 |
| Exon3 | 5'-ATGTGGGGACAGGTTTG-3' | 5'-AAGGGGTCAGTGGAGGT-3' | 356 |
| Exon4-5 | 5'-AAGGCGCTTTCACCAGT-3' | 5'-CATTCTTGGTTAGGAGACATTA-3' | 724 |
| Exon6 | 5'-AGTTGATCCCCAAAATTAA-3' | 5'-AAAGCCAGAAGGGTTCG-3' | 440 |
| Exon7 | 5'-TGGGCAGTCAGATTTTCC-3' | 5'-AGTTCCTGTGAGTCAGACTAC-3' | 488 |
| Exon8-9 | 5'-GTGCACTGGCAGGAGTC-3' | 5'-GTCTGGAGGATGGAGGG-3' | 367 |
| Exon10 | 5'-GTGCTTGAGTTGATCCTGT-3' | 5'-AGGCTTGAGTCTCATGGA-3' | 371 |
| Exon11 | 5'-CTTTGAGTTGTTTCTAGGTTTCC-3' | 5'-TGCATGGCGGTATGGTG-3' | 405 |
| Exon12 | 5'-CGAGGGCCGTCTGCACT-3' | 5'-GGACAAGTCGGAACCATT-3' | 405 |
