# Supplementary material for: Functional Characterization of Two Low-Density Lipoprotein Receptor Gene Mutations in Two Chinese Patients with Familial Hypercholesterolemia
Source: PLoS One. 2014 Mar 26;9(3):e92703. doi: 10.1371/journal.pone.0092703 (PMC3966815; doi:10.1371/journal.pone.0092703)
Supplement: Table S3 — Sequences of oligonucleotide used for the amplification of apoB gene. (DOCX) [file pone.0092703.s003.docx]

Table S3. Sequences of oligonucleotide used for the amplification of *apoB* gene

| Exon | Forward Primer | Reverse Primer | Size of PCR Product (bp) |
| --- | --- | --- | --- |
| Exon26 | 5'-GGAGCAGTTGACCACAAGCTTAGC-3' | 5'-GGTGGCTTTGCTTGTATGTTCTCC-3' | 310 |
